# Retraction Note: Lidocaine inhibits growth, migration and invasion of gastric carcinoma cells by up-regulation of miR-145

**DOI:** 10.1186/s12885-024-12011-4

**Published:** 2024-02-21

**Authors:** Hongyang Sui, Anfeng Lou, Zhisong Li, Jianjun Yang

**Affiliations:** 1 Department of Emergency, The First Affiliated Hospital of Zhengzhou University, 450052 Zhengzhou, China; 2 Department of Anesthesiology, The First Affiliated Hospital of Zhengzhou University, 450052 Zhengzhou, China

**Retraction Note**: ***BMC Cancer*** 19, **233 (2019)**

10.1186/s12885-019-5431-9

The Editors retracted this article because of significant concerns regarding a number of Figures presented in this work, which question the integrity of the data. Specifically, concerns were raised about the flow cytometry plots in Figs. 1D and 4D. The authors were unable to provide raw data when requested. Author Hongyang Sui stated these figures were incorrect and therefore the conclusions of the article are unreliable. Hongyang Sui also stated that the remaining authors were not aware of this submission.

None of the authors have responded to any correspondence from the editor/publisher about this retraction.

